# Bis[*N*,*N*-bis­(1-allyl-1*H*-benzimidazol-2-ylmethyl-κ*N* ^3^)benzyl­amine-κ*N*]cadmium dipicrate

**DOI:** 10.1107/S1600536811019180

**Published:** 2011-05-25

**Authors:** Jing-Kun Yuan, Ying Bai, Fan Kou, Fei Jia, Hui-Lu Wu

**Affiliations:** a School of Chemical and Biological Engineering, Lanzhou Jiaotong University, Lanzhou 730070, People’s Republic of China

## Abstract

The crystal structure of the title compound, [Cd(C_29_H_29_N_5_)_2_](C_6_H_2_N_3_O_7_)_2_, consists of Cd^II^ complex cations and picrate anions. In the complex cation, the Cd^II^ ion is chelated by two bis­(1-allyl­benzimidazol-2-ylmeth­yl)benzyl­amine (babb) ligands in a distorted octa­hedral geometry. Extensive C—H⋯O hydrogen bonding occurs between cations and anions in the crystal structure.

## Related literature

For applications of benzimidazole derivatives, see: Horton *et al.* (2003 ▶). For crystal structures of related picrate compounds, see: Wu *et al.* (2009*a* ▶,*b* ▶); Yun *et al.* (2008 ▶).
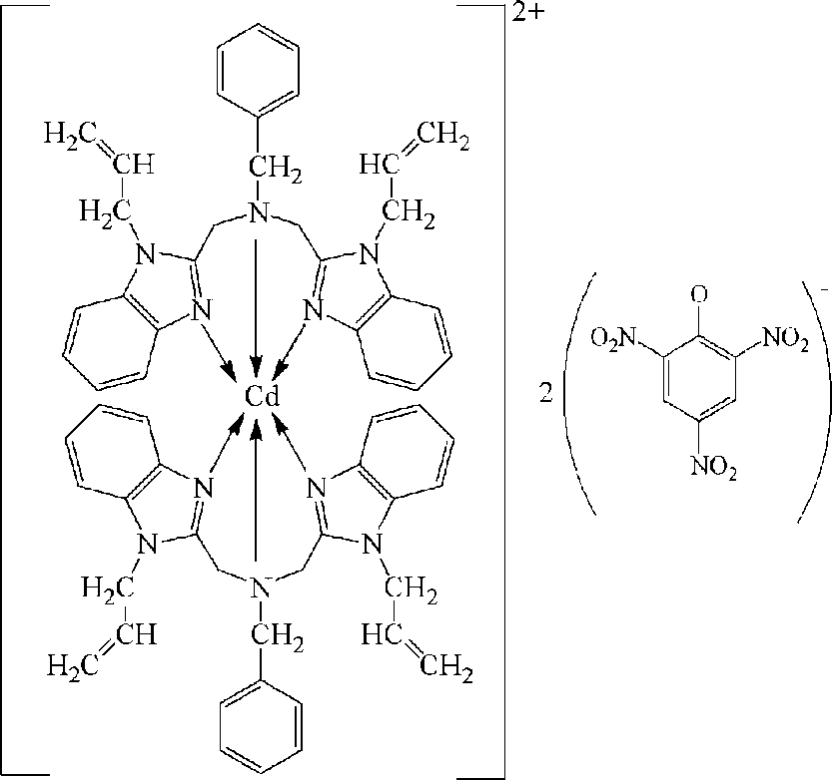


## Experimental

### 

#### Crystal data

[Cd(C_29_H_29_N_5_)_2_](C_6_H_2_N_3_O_7_)_2_
*M* *_r_* = 1463.76
Triclinic, 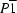

*a* = 13.903 (4) Å
*b* = 14.146 (4) Å
*c* = 19.330 (5) Å
α = 90.595 (3)°
β = 110.890 (3)°
γ = 106.325 (3)°
*V* = 3382.5 (16) Å^3^
*Z* = 2
Mo *K*α radiation
μ = 0.40 mm^−1^
*T* = 296 K
0.40 × 0.38 × 0.30 mm

#### Data collection

Bruker APEXII area-detector diffractometer
Absorption correction: multi-scan (*SADABS*; Sheldrick, 1996 ▶) *T*_min_ = 0.851, *T*_max_ = 0.886
20103 measured reflections
11730 independent reflections
8930 reflections with *I* > 2σ(*I*)
*R*_int_ = 0.027

#### Refinement

*R*[*F* ^2^ > 2σ(*F* ^2^)] = 0.047
*wR*(*F* ^2^) = 0.125
*S* = 1.01
11730 reflections
910 parameters
49 restraints
H-atom parameters constrained
Δρ_max_ = 0.99 e Å^−3^
Δρ_min_ = −0.54 e Å^−3^

### 

Data collection: *APEX2* (Bruker, 2007 ▶); cell refinement: *SAINT* (Bruker, 2007 ▶); data reduction: *SAINT*; program(s) used to solve structure: *SHELXTL* (Sheldrick, 2008 ▶); program(s) used to refine structure: *SHELXTL*; molecular graphics: *SHELXTL*; software used to prepare material for publication: *SHELXTL*.

## Supplementary Material

Crystal structure: contains datablocks global, I. DOI: 10.1107/S1600536811019180/xu5213sup1.cif

Structure factors: contains datablocks I. DOI: 10.1107/S1600536811019180/xu5213Isup2.hkl

Additional supplementary materials:  crystallographic information; 3D view; checkCIF report

## Figures and Tables

**Table 1:** Selected bond lengths (Å)

Cd1—N7	2.556 (3)
Cd1—N9	2.307 (3)
Cd1—N11	2.281 (3)
Cd1—N12	2.673 (3)
Cd1—N13	2.319 (3)
Cd1—N15	2.248 (3)

**Table 2:** Hydrogen-bond geometry (Å, °)

*D*—H⋯*A*	*D*—H	H⋯*A*	*D*⋯*A*	*D*—H⋯*A*
C3—H3⋯O5^i^	0.93	2.49	3.265 (6)	140
C8—H8*A*⋯O10^ii^	0.97	2.60	3.532 (6)	162
C8—H8*B*⋯O4^i^	0.97	2.40	3.363 (6)	171
C21—H21*A*⋯O8^ii^	0.97	2.39	3.277 (7)	151
C21—H21*B*⋯O8	0.97	2.38	3.110 (7)	132
C37—H37*A*⋯O1^iii^	0.97	2.51	3.463 (5)	168
C39—H39*B*⋯O1^iii^	0.97	2.40	3.357 (7)	168
C39—H39*B*⋯O2^iii^	0.97	2.47	3.101 (8)	123
C50—H50*A*⋯O7^iii^	0.97	2.51	3.472 (7)	172
C50—H50*B*⋯O1	0.97	2.28	3.109 (7)	142

Symmetry codes: (i) 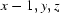; (ii) 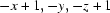; (iii) 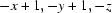.

**Fig. 1.**
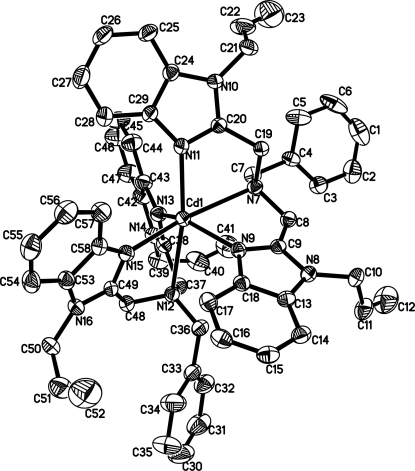
The molecular structure of the title compound. Displacement ellipsoids are drawn at the 30% probability level. H atoms and picrate anion have been omitted for clarity.

## supplementary crystallographic information

### Comment

Benzimidazole derivatives have wide application in medicine, fungicide, biochemical reagents and many other fields (Horton *et al.*, 2003).

In this paper, the cadmium(II) complex with a new type benzimidazole derivative ligand (babb) have been prepared and characterized. Single crystal X-ray diffraction revealed the asymmetric unit of the title compound (Fig. 1) consists of a [Cd^II^(babb)_2_] cation and two picrate anions. In the crystal structure, the Cd(II) ion is hexa-coordinated with two tridentate ligands babb. The shortest Cd–N bond length is 2.248 (3)Å and the longest is 2.672 (3) Å (Table 1). Taking into account the bond length and bond angle, the coordination geometry of the cadmium (II) may be best described as distorted octahedron.

The picrate anions do not coordinate to the metal atom in the structure, similar to those found in the related compounds reported previously (Wu *et al.*, 2009*a*,*b*; Yun *et al.*, 2008). But the extensive C—H···O hydrogen bonding occurs between cation and anion in the crystal structure (Table 2).

### Experimental

To a stirred solution of bis(*N*-allylbenzimidazol-2-ylmethyl)benzylamine (0.2235 g, 0.50 mmol) in room temperature EtOH (5 ml) was added Cd(picrate)_2_ (0.1422 g, 0.25 mmol) solution dissolved in EtOH (5 ml). Owing to the formation of the [Cd^II^(babb)_2_] complex, the yellow precipitate generated immediately. The sediment was filtered off, washed with EtOH and absolute Et_2_O, and dried *in vacuo*. The dried precipitate was dissolved in acetonitrile to a yellow solution that was allowed to evaporate at room temperature. The dried precipitate was dissolved in acetonitrile to a yellow solution and the yellow crystals suitable for X-ray diffraction studies were obtained by ether diffusion into acetonitrile after several days at room temperature. (found: C, 57.77; H, 4.03; N, 15.66. Calcd. for C_70_ H_62_ N_16_ O_14_ Cd: C, 57.44; H, 4.27; N, 15.31)

### Refinement

H atoms were placed in calculated positions with C—H = 0.93 - 0.97 Å, and were refined in a riding-model approximation with *U*_iso_(H) = 1.2*U*_eq_(C). The rigid bond restraints were used for the C21-allyl and C51-allyl groups and for the N3-nitro group.

### Crystal data

**Table:**

[Cd(C_29_H_29_N_5_)_2_](C_6_H_2_N_3_O_7_)_2_	*Z* = 2
*M**_r_* = 1463.76	*F*(000) = 1508
Triclinic, *P*1	*D*_x_ = 1.437 Mg m^−^^3^
Hall symbol: -P 1	Mo *K*α radiation, λ = 0.71073 Å
*a* = 13.903 (4) Å	Cell parameters from 5513 reflections
*b* = 14.146 (4) Å	θ = 2.3–22.5°
*c* = 19.330 (5) Å	µ = 0.40 mm^−^^1^
α = 90.595 (3)°	*T* = 296 K
β = 110.890 (3)°	Block, yellow
γ = 106.325 (3)°	0.40 × 0.38 × 0.30 mm
*V* = 3382.5 (16) Å^3^	

### Data collection

**Table:**

Bruker APEXII area-detector diffractometer	11730 independent reflections
Radiation source: fine-focus sealed tube	8930 reflections with *I* > 2σ(*I*)
graphite	*R*_int_ = 0.027
ω scans	θ_max_ = 25.0°, θ_min_ = 2.3°
Absorption correction: multi-scan (*SADABS*; Sheldrick, 1996)	*h* = −16→16
*T*_min_ = 0.851, *T*_max_ = 0.886	*k* = −15→16
20103 measured reflections	*l* = −22→22

### Refinement

**Table:**

Refinement on *F*^2^	Primary atom site location: structure-invariant direct methods
Least-squares matrix: full	Secondary atom site location: difference Fourier map
*R*[*F*^2^ > 2σ(*F*^2^)] = 0.047	Hydrogen site location: inferred from neighbouring sites
*wR*(*F*^2^) = 0.125	H-atom parameters constrained
*S* = 1.01	*w* = 1/[σ^2^(*F*_o_^2^) + (0.0601*P*)^2^ + 1.9501*P*] where *P* = (*F*_o_^2^ + 2*F*_c_^2^)/3
11730 reflections	(Δ/σ)_max_ = 0.004
910 parameters	Δρ_max_ = 0.99 e Å^−^^3^
49 restraints	Δρ_min_ = −0.54 e Å^−^^3^

### Special details

**Table:**

Geometry. All e.s.d.'s (except the e.s.d. in the dihedral angle between two l.s. planes) are estimated using the full covariance matrix. The cell e.s.d.'s are taken into account individually in the estimation of e.s.d.'s in distances, angles and torsion angles; correlations between e.s.d.'s in cell parameters are only used when they are defined by crystal symmetry. An approximate (isotropic) treatment of cell e.s.d.'s is used for estimating e.s.d.'s involving l.s. planes.
Refinement. Refinement of *F*^2^ against ALL reflections. The weighted *R*-factor *wR* and goodness of fit *S* are based on *F*^2^, conventional *R*-factors *R* are based on *F*, with *F* set to zero for negative *F*^2^. The threshold expression of *F*^2^ > σ(*F*^2^) is used only for calculating *R*-factors(gt) *etc*. and is not relevant to the choice of reflections for refinement. *R*-factors based on *F*^2^ are statistically about twice as large as those based on *F*, and *R*- factors based on ALL data will be even larger.

### Fractional atomic coordinates and isotropic or equivalent isotropic displacement parameters (Å^2^)

**Table:**

	*x*	*y*	*z*	*U*_iso_*/*U*_eq_	
Cd1	0.45078 (2)	0.30077 (2)	0.244601 (15)	0.04046 (11)	
N7	0.3168 (2)	0.1770 (2)	0.28772 (16)	0.0388 (7)	
N8	0.3115 (2)	0.3964 (2)	0.39291 (17)	0.0412 (7)	
N9	0.4218 (2)	0.3794 (2)	0.33711 (16)	0.0378 (7)	
N10	0.5239 (3)	0.0499 (2)	0.35712 (17)	0.0430 (7)	
N11	0.5293 (3)	0.1843 (2)	0.29740 (17)	0.0439 (8)	
N12	0.3534 (2)	0.4120 (2)	0.15000 (17)	0.0415 (7)	
N13	0.3660 (3)	0.2227 (2)	0.12293 (17)	0.0464 (8)	
N14	0.2367 (3)	0.1915 (3)	0.00994 (18)	0.0521 (9)	
N15	0.5770 (3)	0.4316 (2)	0.23197 (18)	0.0441 (8)	
N16	0.6319 (3)	0.5630 (2)	0.17875 (18)	0.0457 (8)	
C1	−0.0145 (6)	−0.1181 (5)	0.2642 (3)	0.092 (2)	
H1	−0.0707	−0.1646	0.2722	0.111*	
C2	−0.0276 (4)	−0.0311 (4)	0.2419 (3)	0.0777 (15)	
H2	−0.0919	−0.0175	0.2350	0.093*	
C3	0.0554 (3)	0.0378 (3)	0.2297 (3)	0.0580 (11)	
H3	0.0454	0.0973	0.2135	0.070*	
C4	0.1526 (3)	0.0213 (3)	0.2405 (2)	0.0448 (9)	
C5	0.1641 (5)	−0.0689 (3)	0.2627 (3)	0.0741 (15)	
H5	0.2280	−0.0832	0.2694	0.089*	
C6	0.0789 (6)	−0.1397 (4)	0.2752 (3)	0.091 (2)	
H6	0.0866	−0.2001	0.2906	0.109*	
C7	0.2409 (3)	0.0982 (3)	0.2259 (2)	0.0475 (10)	
H7A	0.2830	0.0639	0.2107	0.057*	
H7B	0.2070	0.1301	0.1838	0.057*	
C8	0.2604 (3)	0.2338 (3)	0.3154 (2)	0.0439 (9)	
H8A	0.2366	0.1994	0.3523	0.053*	
H8B	0.1971	0.2381	0.2744	0.053*	
C9	0.3336 (3)	0.3359 (3)	0.3495 (2)	0.0375 (8)	
C10	0.2226 (3)	0.3715 (3)	0.4207 (2)	0.0495 (10)	
H10A	0.2464	0.4103	0.4687	0.059*	
H10B	0.2055	0.3020	0.4281	0.059*	
C11	0.1240 (4)	0.3905 (4)	0.3694 (3)	0.0688 (14)	
H11	0.1304	0.4517	0.3513	0.083*	
C12	0.0303 (5)	0.3267 (6)	0.3487 (4)	0.109 (2)	
H12A	0.0219	0.2650	0.3661	0.131*	
H12B	−0.0296	0.3420	0.3163	0.131*	
C13	0.3905 (3)	0.4872 (3)	0.4084 (2)	0.0421 (9)	
C14	0.4055 (3)	0.5761 (3)	0.4479 (2)	0.0516 (10)	
H14	0.3585	0.5828	0.4705	0.062*	
C15	0.4937 (4)	0.6539 (3)	0.4518 (2)	0.0550 (11)	
H15	0.5063	0.7149	0.4775	0.066*	
C16	0.5646 (4)	0.6442 (3)	0.4184 (2)	0.0537 (11)	
H16	0.6232	0.6986	0.4222	0.064*	
C17	0.5499 (3)	0.5553 (3)	0.3797 (2)	0.0485 (10)	
H17	0.5980	0.5486	0.3582	0.058*	
C18	0.4606 (3)	0.4764 (3)	0.3743 (2)	0.0401 (9)	
C19	0.3867 (3)	0.1373 (3)	0.3500 (2)	0.0403 (9)	
H19A	0.3442	0.0745	0.3584	0.048*	
H19B	0.4148	0.1832	0.3953	0.048*	
C20	0.4787 (3)	0.1224 (3)	0.3331 (2)	0.0386 (9)	
C21	0.4891 (4)	−0.0338 (3)	0.3959 (3)	0.0593 (10)	
H21A	0.4391	−0.0213	0.4166	0.071*	
H21B	0.5513	−0.0407	0.4366	0.071*	
C22	0.4337 (5)	−0.1304 (4)	0.3412 (4)	0.0900 (15)	
H22	0.4612	−0.1352	0.3043	0.108*	
C24	0.6123 (3)	0.0667 (3)	0.3361 (2)	0.0455 (9)	
C25	0.6879 (4)	0.0165 (4)	0.3465 (2)	0.0596 (12)	
H25	0.6863	−0.0386	0.3724	0.072*	
C26	0.7650 (4)	0.0519 (4)	0.3170 (3)	0.0709 (14)	
H26	0.8174	0.0205	0.3231	0.085*	
C27	0.7668 (4)	0.1347 (4)	0.2777 (3)	0.0745 (15)	
H27	0.8201	0.1564	0.2581	0.089*	
C28	0.6917 (4)	0.1851 (4)	0.2673 (3)	0.0614 (12)	
H28	0.6929	0.2396	0.2408	0.074*	
C29	0.6147 (3)	0.1504 (3)	0.2982 (2)	0.0457 (10)	
C30	0.2079 (8)	0.7078 (7)	0.0681 (4)	0.113 (3)	
H30	0.1824	0.7573	0.0438	0.135*	
C31	0.1424 (6)	0.6136 (6)	0.0525 (3)	0.096 (2)	
H31	0.0726	0.5984	0.0170	0.115*	
C32	0.1787 (4)	0.5390 (4)	0.0894 (3)	0.0699 (14)	
H32	0.1334	0.4738	0.0775	0.084*	
C33	0.2816 (4)	0.5608 (3)	0.1436 (2)	0.0531 (11)	
C34	0.3468 (5)	0.6565 (4)	0.1576 (4)	0.0915 (18)	
H34	0.4165	0.6727	0.1934	0.110*	
C35	0.3103 (8)	0.7315 (5)	0.1186 (5)	0.124 (3)	
H35	0.3562	0.7963	0.1276	0.148*	
C36	0.3225 (3)	0.4831 (3)	0.1882 (2)	0.0470 (10)	
H36A	0.3850	0.5172	0.2323	0.056*	
H36B	0.2669	0.4453	0.2051	0.056*	
C37	0.2606 (3)	0.3419 (3)	0.0907 (2)	0.0473 (10)	
H37A	0.2408	0.3735	0.0456	0.057*	
H37B	0.1992	0.3232	0.1059	0.057*	
C38	0.2883 (3)	0.2518 (3)	0.0753 (2)	0.0448 (9)	
C39	0.1432 (4)	0.1985 (4)	−0.0532 (2)	0.0641 (13)	
H39A	0.1410	0.1643	−0.0978	0.077*	
H39B	0.1531	0.2679	−0.0604	0.077*	
C40	0.0380 (5)	0.1570 (4)	−0.0455 (4)	0.0874 (17)	
H40	−0.0229	0.1552	−0.0870	0.105*	
C41	0.0210 (5)	0.1240 (5)	0.0098 (4)	0.107 (2)	
H41A	0.0787	0.1238	0.0530	0.128*	
H41B	−0.0495	0.0996	0.0080	0.128*	
C42	0.2839 (4)	0.1160 (3)	0.0158 (2)	0.0543 (11)	
C43	0.2641 (5)	0.0356 (4)	−0.0339 (3)	0.0774 (15)	
H43	0.2087	0.0218	−0.0807	0.093*	
C44	0.3305 (5)	−0.0226 (4)	−0.0106 (4)	0.0920 (18)	
H44	0.3200	−0.0771	−0.0428	0.110*	
C45	0.4128 (5)	−0.0033 (4)	0.0593 (3)	0.0839 (17)	
H45	0.4564	−0.0446	0.0727	0.101*	
C46	0.4312 (4)	0.0761 (4)	0.1092 (3)	0.0664 (13)	
H46	0.4856	0.0888	0.1564	0.080*	
C47	0.3648 (4)	0.1364 (3)	0.0860 (2)	0.0506 (10)	
C48	0.4365 (3)	0.4627 (3)	0.1209 (2)	0.0453 (9)	
H48A	0.4232	0.5233	0.1030	0.054*	
H48B	0.4310	0.4204	0.0790	0.054*	
C49	0.5478 (3)	0.4872 (3)	0.1787 (2)	0.0416 (9)	
C50	0.6307 (4)	0.6398 (3)	0.1282 (3)	0.0663 (12)	
H50A	0.5643	0.6175	0.0844	0.080*	
H50B	0.6905	0.6478	0.1118	0.080*	
C51	0.6388 (5)	0.7379 (4)	0.1622 (4)	0.0915 (16)	
H51	0.6352	0.7871	0.1307	0.110*	
C52	0.6493 (7)	0.7619 (6)	0.2236 (5)	0.142 (3)	
H52A	0.6535	0.7163	0.2580	0.170*	
H52B	0.6533	0.8264	0.2377	0.170*	
C53	0.7238 (3)	0.5547 (3)	0.2355 (2)	0.0491 (10)	
C54	0.8320 (4)	0.6084 (3)	0.2586 (3)	0.0617 (12)	
H54	0.8559	0.6622	0.2353	0.074*	
C55	0.9020 (4)	0.5788 (4)	0.3172 (3)	0.0717 (14)	
H55	0.9755	0.6128	0.3337	0.086*	
C56	0.8670 (4)	0.4994 (4)	0.3531 (3)	0.0695 (14)	
H56	0.9173	0.4825	0.3935	0.083*	
C57	0.7597 (4)	0.4455 (3)	0.3302 (2)	0.0588 (11)	
H57	0.7361	0.3927	0.3546	0.071*	
C58	0.6883 (3)	0.4730 (3)	0.2694 (2)	0.0447 (9)	
C23	0.3603 (7)	−0.1997 (6)	0.3398 (5)	0.142 (3)	
H23A	0.3292	−0.1996	0.3751	0.170*	
H23B	0.3343	−0.2539	0.3034	0.170*	
O1	0.7855 (3)	0.5608 (3)	0.0776 (2)	0.0854 (11)	
O2	0.9959 (4)	0.6717 (4)	0.1386 (4)	0.138 (2)	
O3	1.0943 (4)	0.6156 (3)	0.2283 (3)	0.1176 (16)	
O4	1.0571 (3)	0.2747 (3)	0.1729 (2)	0.0804 (10)	
O5	0.8978 (3)	0.1686 (3)	0.1459 (2)	0.0926 (12)	
O6	0.5905 (4)	0.2801 (5)	0.0524 (5)	0.222 (3)	
O7	0.5958 (3)	0.4218 (3)	0.0364 (2)	0.1058 (14)	
N1	1.0145 (4)	0.6059 (4)	0.1727 (3)	0.0855 (14)	
N2	0.9580 (4)	0.2541 (3)	0.1550 (2)	0.0656 (10)	
N3	0.6417 (4)	0.3631 (4)	0.0590 (3)	0.0883 (14)	
C59	0.8232 (4)	0.4936 (4)	0.1024 (3)	0.0630 (13)	
C60	0.9360 (4)	0.5060 (4)	0.1448 (3)	0.0628 (12)	
C61	0.9780 (4)	0.4297 (4)	0.1627 (3)	0.0624 (12)	
H61	1.0520	0.4426	0.1881	0.075*	
C62	0.9115 (4)	0.3337 (4)	0.1435 (2)	0.0550 (11)	
C63	0.8012 (4)	0.3131 (4)	0.1119 (2)	0.0597 (12)	
H63	0.7563	0.2480	0.1034	0.072*	
C64	0.7581 (4)	0.3899 (4)	0.0931 (2)	0.0619 (12)	
O8	0.6972 (3)	0.0767 (3)	0.5319 (2)	0.0803 (10)	
O9	0.7204 (3)	−0.1041 (3)	0.5219 (2)	0.0869 (11)	
O10	0.8845 (4)	−0.1067 (3)	0.5786 (2)	0.0881 (11)	
O11	1.1825 (3)	0.1588 (3)	0.5937 (2)	0.0918 (12)	
O12	1.1644 (3)	0.3009 (3)	0.5607 (2)	0.0937 (12)	
O13	0.8299 (4)	0.3629 (4)	0.5237 (4)	0.143 (2)	
O14	0.6858 (4)	0.2479 (3)	0.4708 (3)	0.1159 (17)	
N4	0.8182 (4)	−0.0629 (3)	0.5479 (2)	0.0673 (11)	
N5	1.1273 (3)	0.2139 (4)	0.5695 (2)	0.0688 (11)	
N6	0.7818 (4)	0.2757 (4)	0.5033 (3)	0.0785 (13)	
C65	0.7887 (4)	0.1034 (3)	0.5305 (2)	0.0540 (11)	
C70	0.8600 (4)	0.0415 (3)	0.5428 (2)	0.0491 (10)	
C69	0.9672 (4)	0.0775 (3)	0.5553 (2)	0.0506 (10)	
H69	1.0102	0.0356	0.5669	0.061*	
C68	1.0116 (3)	0.1758 (3)	0.5507 (2)	0.0517 (11)	
C67	0.9492 (4)	0.2393 (3)	0.5320 (2)	0.0556 (11)	
H67	0.9796	0.3051	0.5271	0.067*	
C66	0.8422 (4)	0.2037 (3)	0.5211 (2)	0.0560 (11)	

### Atomic displacement parameters (Å^2^)

**Table:**

	*U*^11^	*U*^22^	*U*^33^	*U*^12^	*U*^13^	*U*^23^
Cd1	0.04134 (18)	0.04066 (18)	0.04196 (17)	0.01242 (13)	0.01851 (13)	0.01003 (12)
N7	0.0348 (17)	0.0383 (18)	0.0427 (17)	0.0126 (15)	0.0127 (14)	0.0053 (14)
N8	0.0368 (18)	0.0442 (19)	0.0451 (18)	0.0151 (16)	0.0161 (15)	0.0076 (15)
N9	0.0359 (17)	0.0366 (18)	0.0393 (17)	0.0080 (14)	0.0146 (14)	0.0043 (14)
N10	0.0413 (18)	0.0454 (18)	0.0479 (18)	0.0200 (15)	0.0178 (15)	0.0151 (14)
N11	0.0398 (18)	0.048 (2)	0.0501 (19)	0.0158 (16)	0.0215 (16)	0.0129 (16)
N12	0.0396 (18)	0.0448 (19)	0.0419 (17)	0.0140 (16)	0.0166 (15)	0.0071 (15)
N13	0.054 (2)	0.045 (2)	0.0425 (18)	0.0182 (17)	0.0180 (17)	0.0074 (15)
N14	0.057 (2)	0.044 (2)	0.044 (2)	0.0102 (18)	0.0097 (17)	0.0028 (16)
N15	0.0388 (19)	0.0441 (19)	0.0504 (19)	0.0132 (16)	0.0175 (16)	0.0109 (16)
N16	0.049 (2)	0.0393 (19)	0.0503 (19)	0.0106 (16)	0.0227 (17)	0.0106 (15)
C1	0.100 (5)	0.077 (4)	0.058 (3)	−0.029 (4)	0.025 (3)	−0.007 (3)
C2	0.067 (3)	0.071 (4)	0.081 (4)	−0.006 (3)	0.034 (3)	−0.014 (3)
C3	0.046 (3)	0.049 (3)	0.071 (3)	0.008 (2)	0.018 (2)	0.002 (2)
C4	0.048 (2)	0.034 (2)	0.040 (2)	0.0071 (19)	0.0057 (18)	0.0026 (17)
C5	0.080 (4)	0.047 (3)	0.072 (3)	0.021 (3)	0.000 (3)	0.004 (2)
C6	0.126 (6)	0.037 (3)	0.069 (4)	−0.001 (4)	0.007 (4)	0.015 (2)
C7	0.045 (2)	0.049 (2)	0.043 (2)	0.012 (2)	0.0109 (19)	0.0010 (19)
C8	0.034 (2)	0.045 (2)	0.052 (2)	0.0128 (19)	0.0152 (18)	0.0078 (19)
C9	0.034 (2)	0.041 (2)	0.038 (2)	0.0149 (18)	0.0113 (17)	0.0099 (17)
C10	0.040 (2)	0.058 (3)	0.057 (3)	0.017 (2)	0.024 (2)	0.005 (2)
C11	0.042 (3)	0.087 (4)	0.084 (4)	0.024 (3)	0.027 (3)	0.010 (3)
C12	0.072 (4)	0.150 (7)	0.107 (5)	0.045 (5)	0.025 (4)	0.017 (5)
C13	0.040 (2)	0.044 (2)	0.040 (2)	0.0166 (19)	0.0088 (18)	0.0058 (18)
C14	0.053 (3)	0.053 (3)	0.053 (2)	0.025 (2)	0.019 (2)	0.008 (2)
C15	0.063 (3)	0.043 (3)	0.053 (3)	0.020 (2)	0.012 (2)	0.001 (2)
C16	0.052 (3)	0.044 (3)	0.051 (2)	0.006 (2)	0.009 (2)	0.006 (2)
C17	0.043 (2)	0.049 (3)	0.047 (2)	0.008 (2)	0.0153 (19)	0.0053 (19)
C18	0.039 (2)	0.043 (2)	0.038 (2)	0.0149 (19)	0.0119 (17)	0.0101 (17)
C19	0.039 (2)	0.043 (2)	0.043 (2)	0.0165 (18)	0.0170 (18)	0.0110 (17)
C20	0.033 (2)	0.042 (2)	0.038 (2)	0.0119 (18)	0.0098 (17)	0.0058 (17)
C21	0.055 (2)	0.059 (2)	0.073 (2)	0.0265 (19)	0.028 (2)	0.0325 (19)
C22	0.072 (3)	0.048 (3)	0.148 (4)	0.020 (2)	0.037 (3)	0.034 (3)
C24	0.040 (2)	0.053 (3)	0.044 (2)	0.018 (2)	0.0153 (18)	0.0081 (19)
C25	0.057 (3)	0.074 (3)	0.062 (3)	0.039 (3)	0.022 (2)	0.017 (2)
C26	0.057 (3)	0.087 (4)	0.086 (4)	0.043 (3)	0.031 (3)	0.015 (3)
C27	0.059 (3)	0.093 (4)	0.087 (4)	0.028 (3)	0.042 (3)	0.013 (3)
C28	0.055 (3)	0.069 (3)	0.073 (3)	0.022 (2)	0.035 (2)	0.020 (2)
C29	0.041 (2)	0.054 (3)	0.050 (2)	0.022 (2)	0.0207 (19)	0.012 (2)
C30	0.176 (9)	0.117 (6)	0.097 (5)	0.103 (7)	0.065 (6)	0.050 (5)
C31	0.113 (5)	0.131 (6)	0.066 (4)	0.082 (5)	0.027 (4)	0.025 (4)
C32	0.071 (3)	0.085 (4)	0.062 (3)	0.041 (3)	0.020 (3)	0.012 (3)
C33	0.057 (3)	0.058 (3)	0.055 (3)	0.028 (2)	0.025 (2)	0.007 (2)
C34	0.087 (4)	0.061 (4)	0.126 (5)	0.030 (3)	0.034 (4)	0.018 (3)
C35	0.167 (8)	0.069 (4)	0.162 (8)	0.055 (5)	0.077 (7)	0.042 (5)
C36	0.051 (2)	0.052 (3)	0.043 (2)	0.023 (2)	0.0171 (19)	0.0047 (19)
C37	0.040 (2)	0.053 (3)	0.047 (2)	0.014 (2)	0.0140 (19)	0.0076 (19)
C38	0.047 (2)	0.046 (2)	0.041 (2)	0.012 (2)	0.0171 (19)	0.0103 (19)
C39	0.068 (3)	0.056 (3)	0.050 (3)	0.014 (3)	0.005 (2)	0.002 (2)
C40	0.080 (4)	0.069 (4)	0.083 (4)	0.006 (3)	0.007 (3)	0.012 (3)
C41	0.081 (4)	0.090 (5)	0.126 (6)	0.006 (4)	0.026 (4)	0.017 (4)
C42	0.061 (3)	0.041 (2)	0.054 (3)	0.009 (2)	0.019 (2)	0.004 (2)
C43	0.096 (4)	0.057 (3)	0.064 (3)	0.022 (3)	0.015 (3)	−0.009 (3)
C44	0.117 (5)	0.056 (3)	0.093 (4)	0.026 (4)	0.028 (4)	−0.018 (3)
C45	0.101 (5)	0.053 (3)	0.100 (4)	0.034 (3)	0.032 (4)	0.004 (3)
C46	0.076 (3)	0.059 (3)	0.065 (3)	0.028 (3)	0.021 (3)	0.003 (2)
C47	0.060 (3)	0.043 (2)	0.051 (2)	0.016 (2)	0.022 (2)	0.008 (2)
C48	0.047 (2)	0.048 (2)	0.047 (2)	0.018 (2)	0.023 (2)	0.0125 (19)
C49	0.045 (2)	0.037 (2)	0.052 (2)	0.0124 (19)	0.027 (2)	0.0089 (18)
C50	0.071 (3)	0.051 (2)	0.092 (3)	0.021 (2)	0.046 (3)	0.030 (2)
C51	0.089 (3)	0.059 (3)	0.126 (4)	0.016 (3)	0.046 (3)	0.015 (3)
C52	0.142 (6)	0.100 (5)	0.157 (6)	0.013 (5)	0.044 (6)	−0.030 (5)
C53	0.049 (3)	0.040 (2)	0.057 (3)	0.004 (2)	0.025 (2)	0.0004 (19)
C54	0.050 (3)	0.056 (3)	0.073 (3)	0.000 (2)	0.028 (3)	0.000 (2)
C55	0.046 (3)	0.080 (4)	0.075 (3)	0.003 (3)	0.019 (3)	−0.010 (3)
C56	0.051 (3)	0.083 (4)	0.063 (3)	0.022 (3)	0.008 (2)	0.001 (3)
C57	0.051 (3)	0.063 (3)	0.059 (3)	0.017 (2)	0.016 (2)	0.011 (2)
C58	0.041 (2)	0.047 (2)	0.047 (2)	0.012 (2)	0.0188 (19)	0.0058 (19)
C23	0.124 (6)	0.100 (5)	0.189 (7)	0.017 (4)	0.058 (5)	0.018 (5)
O1	0.100 (3)	0.086 (3)	0.105 (3)	0.062 (2)	0.052 (2)	0.046 (2)
O2	0.120 (4)	0.076 (3)	0.204 (6)	0.035 (3)	0.042 (4)	0.042 (4)
O3	0.095 (3)	0.097 (3)	0.131 (4)	0.017 (3)	0.018 (3)	−0.003 (3)
O4	0.063 (2)	0.085 (3)	0.099 (3)	0.040 (2)	0.023 (2)	0.026 (2)
O5	0.080 (3)	0.061 (2)	0.133 (4)	0.030 (2)	0.029 (2)	0.024 (2)
O6	0.065 (3)	0.138 (4)	0.405 (8)	0.022 (3)	0.025 (4)	0.103 (6)
O7	0.081 (3)	0.126 (3)	0.107 (3)	0.061 (3)	0.008 (2)	0.019 (3)
N1	0.086 (4)	0.071 (3)	0.111 (4)	0.037 (3)	0.040 (3)	0.015 (3)
N2	0.063 (3)	0.069 (3)	0.066 (3)	0.031 (2)	0.017 (2)	0.015 (2)
N3	0.060 (3)	0.100 (3)	0.113 (3)	0.037 (2)	0.032 (3)	0.040 (3)
C59	0.075 (3)	0.072 (3)	0.064 (3)	0.040 (3)	0.038 (3)	0.023 (3)
C60	0.068 (3)	0.057 (3)	0.075 (3)	0.026 (3)	0.034 (3)	0.013 (2)
C61	0.056 (3)	0.074 (3)	0.063 (3)	0.030 (3)	0.022 (2)	0.012 (3)
C62	0.056 (3)	0.063 (3)	0.054 (3)	0.030 (3)	0.021 (2)	0.019 (2)
C63	0.062 (3)	0.065 (3)	0.060 (3)	0.028 (3)	0.026 (2)	0.018 (2)
C64	0.056 (3)	0.084 (4)	0.057 (3)	0.035 (3)	0.024 (2)	0.020 (3)
O8	0.050 (2)	0.096 (3)	0.094 (3)	0.0109 (19)	0.0347 (19)	−0.008 (2)
O9	0.076 (3)	0.071 (2)	0.104 (3)	−0.009 (2)	0.046 (2)	0.005 (2)
O10	0.113 (3)	0.063 (2)	0.093 (3)	0.029 (2)	0.041 (2)	0.026 (2)
O11	0.059 (2)	0.113 (3)	0.110 (3)	0.035 (2)	0.032 (2)	0.018 (3)
O12	0.081 (3)	0.083 (3)	0.126 (3)	0.003 (2)	0.065 (3)	0.012 (2)
O13	0.106 (4)	0.076 (3)	0.221 (6)	0.042 (3)	0.021 (4)	−0.006 (4)
O14	0.086 (3)	0.120 (4)	0.118 (4)	0.059 (3)	−0.010 (3)	−0.025 (3)
N4	0.083 (3)	0.061 (3)	0.063 (3)	0.014 (3)	0.039 (2)	0.011 (2)
N5	0.061 (3)	0.078 (3)	0.074 (3)	0.011 (3)	0.040 (2)	0.004 (2)
N6	0.075 (3)	0.077 (3)	0.077 (3)	0.037 (3)	0.012 (3)	−0.007 (3)
C65	0.047 (3)	0.067 (3)	0.047 (2)	0.012 (2)	0.021 (2)	−0.002 (2)
C70	0.059 (3)	0.045 (2)	0.044 (2)	0.008 (2)	0.026 (2)	0.0040 (19)
C69	0.056 (3)	0.058 (3)	0.045 (2)	0.021 (2)	0.026 (2)	0.009 (2)
C68	0.050 (3)	0.061 (3)	0.049 (2)	0.013 (2)	0.029 (2)	0.004 (2)
C67	0.068 (3)	0.050 (3)	0.053 (3)	0.015 (2)	0.029 (2)	0.008 (2)
C66	0.061 (3)	0.057 (3)	0.054 (3)	0.026 (2)	0.020 (2)	0.001 (2)

### Geometric parameters (Å, °)

**Table:**

Cd1—N7	2.556 (3)	C31—H31	0.9300
Cd1—N9	2.307 (3)	C32—C33	1.384 (6)
Cd1—N11	2.281 (3)	C32—H32	0.9300
Cd1—N12	2.673 (3)	C33—C34	1.362 (7)
Cd1—N13	2.319 (3)	C33—C36	1.516 (6)
Cd1—N15	2.248 (3)	C34—C35	1.413 (9)
N7—C8	1.480 (5)	C34—H34	0.9300
N7—C19	1.482 (4)	C35—H35	0.9300
N7—C7	1.482 (5)	C36—H36A	0.9700
N8—C9	1.360 (5)	C36—H36B	0.9700
N8—C13	1.382 (5)	C37—C38	1.487 (5)
N8—C10	1.474 (5)	C37—H37A	0.9700
N9—C9	1.315 (4)	C37—H37B	0.9700
N9—C18	1.406 (5)	C39—C40	1.478 (8)
N10—C20	1.352 (4)	C39—H39A	0.9700
N10—C24	1.388 (5)	C39—H39B	0.9700
N10—C21	1.467 (5)	C40—C41	1.243 (8)
N11—C20	1.323 (5)	C40—H40	0.9300
N11—C29	1.394 (5)	C41—H41A	0.9300
N12—C37	1.471 (5)	C41—H41B	0.9300
N12—C48	1.472 (5)	C42—C43	1.380 (6)
N12—C36	1.481 (5)	C42—C47	1.383 (6)
N13—C38	1.316 (5)	C43—C44	1.367 (7)
N13—C47	1.401 (5)	C43—H43	0.9300
N14—C38	1.351 (5)	C44—C45	1.388 (8)
N14—C42	1.389 (5)	C44—H44	0.9300
N14—C39	1.461 (5)	C45—C46	1.377 (7)
N15—C49	1.319 (5)	C45—H45	0.9300
N15—C58	1.394 (5)	C46—C47	1.391 (6)
N16—C49	1.346 (5)	C46—H46	0.9300
N16—C53	1.391 (5)	C48—C49	1.491 (5)
N16—C50	1.468 (5)	C48—H48A	0.9700
C1—C2	1.348 (8)	C48—H48B	0.9700
C1—C6	1.361 (9)	C50—C51	1.487 (7)
C1—H1	0.9300	C50—H50A	0.9700
C2—C3	1.380 (7)	C50—H50B	0.9700
C2—H2	0.9300	C51—C52	1.177 (9)
C3—C4	1.379 (6)	C51—H51	0.9300
C3—H3	0.9300	C52—H52A	0.9300
C4—C5	1.384 (6)	C52—H52B	0.9300
C4—C7	1.509 (6)	C53—C54	1.381 (6)
C5—C6	1.417 (8)	C53—C58	1.391 (6)
C5—H5	0.9300	C54—C55	1.363 (7)
C6—H6	0.9300	C54—H54	0.9300
C7—H7A	0.9700	C55—C56	1.389 (7)
C7—H7B	0.9700	C55—H55	0.9300
C8—C9	1.494 (5)	C56—C57	1.373 (6)
C8—H8A	0.9700	C56—H56	0.9300
C8—H8B	0.9700	C57—C58	1.385 (6)
C10—C11	1.475 (6)	C57—H57	0.9300
C10—H10A	0.9700	C23—H23A	0.9300
C10—H10B	0.9700	C23—H23B	0.9300
C11—C12	1.278 (8)	O1—C59	1.232 (5)
C11—H11	0.9300	O2—N1	1.182 (6)
C12—H12A	0.9300	O3—N1	1.211 (6)
C12—H12B	0.9300	O4—N2	1.240 (5)
C13—C14	1.388 (5)	O5—N2	1.231 (5)
C13—C18	1.397 (5)	O6—N3	1.167 (7)
C14—C15	1.376 (6)	O7—N3	1.181 (5)
C14—H14	0.9300	N1—C60	1.475 (7)
C15—C16	1.392 (6)	N2—C62	1.430 (6)
C15—H15	0.9300	N3—C64	1.444 (6)
C16—C17	1.381 (6)	C59—C60	1.445 (7)
C16—H16	0.9300	C59—C64	1.462 (7)
C17—C18	1.386 (5)	C60—C61	1.359 (6)
C17—H17	0.9300	C61—C62	1.370 (6)
C19—C20	1.495 (5)	C61—H61	0.9300
C19—H19A	0.9700	C62—C63	1.374 (6)
C19—H19B	0.9700	C63—C64	1.375 (6)
C21—C22	1.541 (7)	C63—H63	0.9300
C21—H21A	0.9700	O8—C65	1.232 (5)
C21—H21B	0.9700	O9—N4	1.226 (5)
C22—C23	1.192 (8)	O10—N4	1.234 (5)
C22—H22	0.9300	O11—N5	1.226 (5)
C24—C25	1.386 (5)	O12—N5	1.233 (5)
C24—C29	1.398 (5)	O13—N6	1.209 (6)
C25—C26	1.366 (6)	O14—N6	1.197 (6)
C25—H25	0.9300	N4—C70	1.447 (6)
C26—C27	1.400 (7)	N5—C68	1.450 (6)
C26—H26	0.9300	N6—C66	1.463 (6)
C27—C28	1.382 (6)	C65—C66	1.452 (6)
C27—H27	0.9300	C65—C70	1.459 (6)
C28—C29	1.382 (6)	C70—C69	1.362 (6)
C28—H28	0.9300	C69—C68	1.371 (6)
C30—C31	1.344 (10)	C69—H69	0.9300
C30—C35	1.352 (10)	C68—C67	1.382 (6)
C30—H30	0.9300	C67—C66	1.365 (6)
C31—C32	1.391 (7)	C67—H67	0.9300
			
N15—Cd1—N11	109.41 (11)	C32—C31—H31	119.9
N15—Cd1—N9	96.74 (11)	C33—C32—C31	120.6 (6)
N11—Cd1—N9	106.65 (11)	C33—C32—H32	119.7
N15—Cd1—N13	100.72 (12)	C31—C32—H32	119.7
N11—Cd1—N13	98.24 (11)	C34—C33—C32	117.9 (5)
N9—Cd1—N13	142.69 (11)	C34—C33—C36	119.5 (4)
N15—Cd1—N7	166.37 (10)	C32—C33—C36	122.5 (4)
N11—Cd1—N7	72.40 (10)	C33—C34—C35	121.2 (6)
N9—Cd1—N7	70.12 (10)	C33—C34—H34	119.4
N13—Cd1—N7	92.25 (11)	C35—C34—H34	119.4
N15—Cd1—N12	70.80 (11)	C30—C35—C34	119.0 (7)
N11—Cd1—N12	165.10 (10)	C30—C35—H35	120.5
N9—Cd1—N12	87.96 (10)	C34—C35—H35	120.5
N13—Cd1—N12	67.50 (10)	N12—C36—C33	117.1 (3)
N7—Cd1—N12	111.11 (9)	N12—C36—H36A	108.0
C8—N7—C19	110.5 (3)	C33—C36—H36A	108.0
C8—N7—C7	112.3 (3)	N12—C36—H36B	108.0
C19—N7—C7	112.1 (3)	C33—C36—H36B	108.0
C8—N7—Cd1	107.3 (2)	H36A—C36—H36B	107.3
C19—N7—Cd1	103.7 (2)	N12—C37—C38	110.2 (3)
C7—N7—Cd1	110.5 (2)	N12—C37—H37A	109.6
C9—N8—C13	107.1 (3)	C38—C37—H37A	109.6
C9—N8—C10	127.6 (3)	N12—C37—H37B	109.6
C13—N8—C10	125.3 (3)	C38—C37—H37B	109.6
C9—N9—C18	105.7 (3)	H37A—C37—H37B	108.1
C9—N9—Cd1	116.9 (2)	N13—C38—N14	112.7 (4)
C18—N9—Cd1	135.3 (2)	N13—C38—C37	124.1 (4)
C20—N10—C24	106.6 (3)	N14—C38—C37	123.2 (4)
C20—N10—C21	129.2 (3)	N14—C39—C40	115.0 (4)
C24—N10—C21	124.2 (3)	N14—C39—H39A	108.5
C20—N11—C29	105.5 (3)	C40—C39—H39A	108.5
C20—N11—Cd1	116.1 (2)	N14—C39—H39B	108.5
C29—N11—Cd1	138.1 (3)	C40—C39—H39B	108.5
C37—N12—C48	110.5 (3)	H39A—C39—H39B	107.5
C37—N12—C36	113.3 (3)	C41—C40—C39	127.8 (6)
C48—N12—C36	112.1 (3)	C41—C40—H40	116.1
C37—N12—Cd1	105.9 (2)	C39—C40—H40	116.1
C48—N12—Cd1	102.6 (2)	C40—C41—H41A	120.0
C36—N12—Cd1	111.8 (2)	C40—C41—H41B	120.0
C38—N13—C47	105.3 (3)	H41A—C41—H41B	120.0
C38—N13—Cd1	119.3 (3)	C43—C42—C47	122.4 (4)
C47—N13—Cd1	134.7 (3)	C43—C42—N14	131.9 (4)
C38—N14—C42	107.1 (3)	C47—C42—N14	105.7 (4)
C38—N14—C39	127.1 (4)	C44—C43—C42	116.4 (5)
C42—N14—C39	125.6 (4)	C44—C43—H43	121.8
C49—N15—C58	106.2 (3)	C42—C43—H43	121.8
C49—N15—Cd1	119.2 (3)	C43—C44—C45	122.4 (5)
C58—N15—Cd1	134.6 (3)	C43—C44—H44	118.8
C49—N16—C53	107.2 (3)	C45—C44—H44	118.8
C49—N16—C50	127.8 (4)	C46—C45—C44	121.1 (5)
C53—N16—C50	125.0 (4)	C46—C45—H45	119.5
C2—C1—C6	121.6 (6)	C44—C45—H45	119.5
C2—C1—H1	119.2	C45—C46—C47	117.2 (5)
C6—C1—H1	119.2	C45—C46—H46	121.4
C1—C2—C3	119.3 (6)	C47—C46—H46	121.4
C1—C2—H2	120.3	C42—C47—C46	120.6 (4)
C3—C2—H2	120.3	C42—C47—N13	109.2 (4)
C4—C3—C2	122.2 (5)	C46—C47—N13	130.2 (4)
C4—C3—H3	118.9	N12—C48—C49	112.2 (3)
C2—C3—H3	118.9	N12—C48—H48A	109.2
C3—C4—C5	117.6 (4)	C49—C48—H48A	109.2
C3—C4—C7	120.4 (4)	N12—C48—H48B	109.2
C5—C4—C7	121.9 (4)	C49—C48—H48B	109.2
C4—C5—C6	120.2 (5)	H48A—C48—H48B	107.9
C4—C5—H5	119.9	N15—C49—N16	112.3 (3)
C6—C5—H5	119.9	N15—C49—C48	123.4 (3)
C1—C6—C5	119.1 (5)	N16—C49—C48	124.1 (3)
C1—C6—H6	120.5	N16—C50—C51	113.9 (4)
C5—C6—H6	120.5	N16—C50—H50A	108.8
N7—C7—C4	117.5 (3)	C51—C50—H50A	108.8
N7—C7—H7A	107.9	N16—C50—H50B	108.8
C4—C7—H7A	107.9	C51—C50—H50B	108.8
N7—C7—H7B	107.9	H50A—C50—H50B	107.7
C4—C7—H7B	107.9	C52—C51—C50	128.9 (8)
H7A—C7—H7B	107.2	C52—C51—H51	115.6
N7—C8—C9	110.7 (3)	C50—C51—H51	115.6
N7—C8—H8A	109.5	C51—C52—H52A	120.0
C9—C8—H8A	109.5	C51—C52—H52B	120.0
N7—C8—H8B	109.5	H52A—C52—H52B	120.0
C9—C8—H8B	109.5	C54—C53—C58	121.6 (4)
H8A—C8—H8B	108.1	C54—C53—N16	132.6 (4)
N9—C9—N8	112.7 (3)	C58—C53—N16	105.9 (3)
N9—C9—C8	124.2 (3)	C55—C54—C53	116.9 (5)
N8—C9—C8	123.0 (3)	C55—C54—H54	121.5
N8—C10—C11	112.9 (4)	C53—C54—H54	121.5
N8—C10—H10A	109.0	C54—C55—C56	122.0 (5)
C11—C10—H10A	109.0	C54—C55—H55	119.0
N8—C10—H10B	109.0	C56—C55—H55	119.0
C11—C10—H10B	109.0	C57—C56—C55	121.4 (5)
H10A—C10—H10B	107.8	C57—C56—H56	119.3
C12—C11—C10	123.0 (6)	C55—C56—H56	119.3
C12—C11—H11	118.5	C56—C57—C58	117.0 (4)
C10—C11—H11	118.5	C56—C57—H57	121.5
C11—C12—H12A	120.0	C58—C57—H57	121.5
C11—C12—H12B	120.0	C57—C58—C53	121.0 (4)
H12A—C12—H12B	120.0	C57—C58—N15	130.6 (4)
N8—C13—C14	131.7 (4)	C53—C58—N15	108.5 (4)
N8—C13—C18	106.1 (3)	C22—C23—H23A	120.0
C14—C13—C18	122.2 (4)	C22—C23—H23B	120.0
C15—C14—C13	116.5 (4)	H23A—C23—H23B	120.0
C15—C14—H14	121.7	O2—N1—O3	123.9 (6)
C13—C14—H14	121.7	O2—N1—C60	117.5 (6)
C14—C15—C16	121.9 (4)	O3—N1—C60	118.6 (5)
C14—C15—H15	119.0	O5—N2—O4	123.1 (4)
C16—C15—H15	119.0	O5—N2—C62	118.7 (4)
C17—C16—C15	121.4 (4)	O4—N2—C62	118.2 (4)
C17—C16—H16	119.3	O6—N3—O7	118.3 (6)
C15—C16—H16	119.3	O6—N3—C64	119.1 (5)
C16—C17—C18	117.5 (4)	O7—N3—C64	122.5 (5)
C16—C17—H17	121.2	O1—C59—C60	125.3 (5)
C18—C17—H17	121.2	O1—C59—C64	123.8 (5)
C17—C18—C13	120.4 (4)	C60—C59—C64	110.9 (4)
C17—C18—N9	131.1 (3)	C61—C60—C59	124.2 (5)
C13—C18—N9	108.5 (3)	C61—C60—N1	115.0 (5)
N7—C19—C20	110.9 (3)	C59—C60—N1	120.8 (4)
N7—C19—H19A	109.5	C60—C61—C62	120.1 (4)
C20—C19—H19A	109.5	C60—C61—H61	120.0
N7—C19—H19B	109.5	C62—C61—H61	120.0
C20—C19—H19B	109.5	C61—C62—C63	120.9 (4)
H19A—C19—H19B	108.0	C61—C62—N2	119.4 (4)
N11—C20—N10	113.0 (3)	C63—C62—N2	119.8 (5)
N11—C20—C19	122.7 (3)	C62—C63—C64	119.2 (5)
N10—C20—C19	124.1 (3)	C62—C63—H63	120.4
N10—C21—C22	110.1 (4)	C64—C63—H63	120.4
N10—C21—H21A	109.6	C63—C64—N3	116.4 (5)
C22—C21—H21A	109.6	C63—C64—C59	123.7 (4)
N10—C21—H21B	109.6	N3—C64—C59	119.8 (4)
C22—C21—H21B	109.6	O9—N4—O10	123.0 (5)
H21A—C21—H21B	108.2	O9—N4—C70	119.7 (5)
C23—C22—C21	128.6 (8)	O10—N4—C70	117.4 (4)
C23—C22—H22	115.7	O11—N5—O12	123.8 (4)
C21—C22—H22	115.7	O11—N5—C68	118.7 (4)
C25—C24—N10	131.6 (4)	O12—N5—C68	117.4 (5)
C25—C24—C29	122.3 (4)	O14—N6—O13	120.8 (5)
N10—C24—C29	106.2 (3)	O14—N6—C66	120.2 (5)
C26—C25—C24	116.8 (4)	O13—N6—C66	119.0 (5)
C26—C25—H25	121.6	O8—C65—C66	123.7 (4)
C24—C25—H25	121.6	O8—C65—C70	125.1 (4)
C25—C26—C27	121.4 (4)	C66—C65—C70	111.2 (4)
C25—C26—H26	119.3	C69—C70—N4	116.9 (4)
C27—C26—H26	119.3	C69—C70—C65	123.8 (4)
C28—C27—C26	122.1 (4)	N4—C70—C65	119.2 (4)
C28—C27—H27	119.0	C70—C69—C68	119.7 (4)
C26—C27—H27	119.0	C70—C69—H69	120.1
C29—C28—C27	116.7 (4)	C68—C69—H69	120.1
C29—C28—H28	121.6	C69—C68—C67	121.4 (4)
C27—C28—H28	121.6	C69—C68—N5	118.7 (4)
C28—C29—N11	130.5 (4)	C67—C68—N5	119.9 (4)
C28—C29—C24	120.8 (4)	C66—C67—C68	118.9 (4)
N11—C29—C24	108.7 (3)	C66—C67—H67	120.6
C31—C30—C35	120.9 (7)	C68—C67—H67	120.6
C31—C30—H30	119.5	C67—C66—C65	124.4 (4)
C35—C30—H30	119.5	C67—C66—N6	115.9 (4)
C30—C31—C32	120.3 (6)	C65—C66—N6	119.6 (4)
C30—C31—H31	119.9		
			
N15—Cd1—N7—C8	43.7 (5)	Cd1—N11—C29—C28	5.1 (7)
N11—Cd1—N7—C8	143.5 (2)	C20—N11—C29—C24	−0.3 (4)
N9—Cd1—N7—C8	27.8 (2)	Cd1—N11—C29—C24	−173.1 (3)
N13—Cd1—N7—C8	−118.6 (2)	C25—C24—C29—C28	2.0 (7)
N12—Cd1—N7—C8	−51.9 (2)	N10—C24—C29—C28	−177.7 (4)
N15—Cd1—N7—C19	−73.3 (5)	C25—C24—C29—N11	−179.6 (4)
N11—Cd1—N7—C19	26.6 (2)	N10—C24—C29—N11	0.8 (4)
N9—Cd1—N7—C19	−89.2 (2)	C35—C30—C31—C32	1.1 (11)
N13—Cd1—N7—C19	124.5 (2)	C30—C31—C32—C33	1.4 (9)
N12—Cd1—N7—C19	−168.8 (2)	C31—C32—C33—C34	−2.2 (7)
N15—Cd1—N7—C7	166.4 (4)	C31—C32—C33—C36	176.5 (4)
N11—Cd1—N7—C7	−93.8 (2)	C32—C33—C34—C35	0.7 (8)
N9—Cd1—N7—C7	150.5 (2)	C36—C33—C34—C35	−178.1 (5)
N13—Cd1—N7—C7	4.1 (2)	C31—C30—C35—C34	−2.7 (12)
N12—Cd1—N7—C7	70.8 (2)	C33—C34—C35—C30	1.8 (11)
N15—Cd1—N9—C9	164.0 (3)	C37—N12—C36—C33	−69.1 (5)
N11—Cd1—N9—C9	−83.4 (3)	C48—N12—C36—C33	56.7 (5)
N13—Cd1—N9—C9	46.3 (3)	Cd1—N12—C36—C33	171.3 (3)
N7—Cd1—N9—C9	−19.8 (2)	C34—C33—C36—N12	−105.4 (5)
N12—Cd1—N9—C9	93.6 (3)	C32—C33—C36—N12	75.9 (5)
N15—Cd1—N9—C18	3.0 (3)	C48—N12—C37—C38	75.2 (4)
N11—Cd1—N9—C18	115.7 (3)	C36—N12—C37—C38	−158.1 (3)
N13—Cd1—N9—C18	−114.6 (3)	Cd1—N12—C37—C38	−35.3 (3)
N7—Cd1—N9—C18	179.3 (3)	C47—N13—C38—N14	−0.3 (4)
N12—Cd1—N9—C18	−67.3 (3)	Cd1—N13—C38—N14	−171.6 (2)
N15—Cd1—N11—C20	155.9 (3)	C47—N13—C38—C37	−179.4 (4)
N9—Cd1—N11—C20	52.3 (3)	Cd1—N13—C38—C37	9.2 (5)
N13—Cd1—N11—C20	−99.6 (3)	C42—N14—C38—N13	0.7 (5)
N7—Cd1—N11—C20	−9.9 (3)	C39—N14—C38—N13	176.2 (4)
N12—Cd1—N11—C20	−115.9 (4)	C42—N14—C38—C37	179.9 (4)
N15—Cd1—N11—C29	−31.8 (4)	C39—N14—C38—C37	−4.6 (6)
N9—Cd1—N11—C29	−135.4 (4)	N12—C37—C38—N13	22.1 (5)
N13—Cd1—N11—C29	72.7 (4)	N12—C37—C38—N14	−157.0 (4)
N7—Cd1—N11—C29	162.4 (4)	C38—N14—C39—C40	−80.3 (6)
N12—Cd1—N11—C29	56.3 (6)	C42—N14—C39—C40	94.4 (5)
N15—Cd1—N12—C37	141.0 (2)	N14—C39—C40—C41	5.7 (9)
N11—Cd1—N12—C37	47.6 (5)	C38—N14—C42—C43	−179.2 (5)
N9—Cd1—N12—C37	−121.2 (2)	C39—N14—C42—C43	5.2 (8)
N13—Cd1—N12—C37	30.0 (2)	C38—N14—C42—C47	−0.9 (5)
N7—Cd1—N12—C37	−53.4 (2)	C39—N14—C42—C47	−176.5 (4)
N15—Cd1—N12—C48	25.1 (2)	C47—C42—C43—C44	−1.1 (8)
N11—Cd1—N12—C48	−68.3 (5)	N14—C42—C43—C44	177.0 (5)
N9—Cd1—N12—C48	122.9 (2)	C42—C43—C44—C45	0.6 (9)
N13—Cd1—N12—C48	−85.9 (2)	C43—C44—C45—C46	0.5 (10)
N7—Cd1—N12—C48	−169.3 (2)	C44—C45—C46—C47	−0.9 (8)
N15—Cd1—N12—C36	−95.2 (3)	C43—C42—C47—C46	0.7 (7)
N11—Cd1—N12—C36	171.4 (4)	N14—C42—C47—C46	−177.9 (4)
N9—Cd1—N12—C36	2.7 (2)	C43—C42—C47—N13	179.3 (4)
N13—Cd1—N12—C36	153.9 (3)	N14—C42—C47—N13	0.8 (5)
N7—Cd1—N12—C36	70.4 (3)	C45—C46—C47—C42	0.4 (7)
N15—Cd1—N13—C38	−85.0 (3)	C45—C46—C47—N13	−177.9 (5)
N11—Cd1—N13—C38	163.3 (3)	C38—N13—C47—C42	−0.3 (5)
N9—Cd1—N13—C38	31.4 (4)	Cd1—N13—C47—C42	169.0 (3)
N7—Cd1—N13—C38	90.8 (3)	C38—N13—C47—C46	178.1 (5)
N12—Cd1—N13—C38	−21.2 (3)	Cd1—N13—C47—C46	−12.5 (7)
N15—Cd1—N13—C47	106.8 (4)	C37—N12—C48—C49	−148.0 (3)
N11—Cd1—N13—C47	−4.9 (4)	C36—N12—C48—C49	84.7 (4)
N9—Cd1—N13—C47	−136.8 (3)	Cd1—N12—C48—C49	−35.4 (3)
N7—Cd1—N13—C47	−77.4 (4)	C58—N15—C49—N16	−0.5 (4)
N12—Cd1—N13—C47	170.6 (4)	Cd1—N15—C49—N16	179.4 (2)
N11—Cd1—N15—C49	153.1 (3)	C58—N15—C49—C48	173.9 (3)
N9—Cd1—N15—C49	−96.6 (3)	Cd1—N15—C49—C48	−6.3 (5)
N13—Cd1—N15—C49	50.3 (3)	C53—N16—C49—N15	1.5 (4)
N7—Cd1—N15—C49	−111.6 (5)	C50—N16—C49—N15	−180.0 (4)
N12—Cd1—N15—C49	−11.1 (3)	C53—N16—C49—C48	−172.8 (3)
N11—Cd1—N15—C58	−27.1 (4)	C50—N16—C49—C48	5.7 (6)
N9—Cd1—N15—C58	83.2 (4)	N12—C48—C49—N15	33.1 (5)
N13—Cd1—N15—C58	−129.9 (3)	N12—C48—C49—N16	−153.1 (3)
N7—Cd1—N15—C58	68.2 (6)	C49—N16—C50—C51	104.0 (5)
N12—Cd1—N15—C58	168.7 (4)	C53—N16—C50—C51	−77.7 (6)
C6—C1—C2—C3	0.3 (8)	N16—C50—C51—C52	2.6 (11)
C1—C2—C3—C4	−1.1 (7)	C49—N16—C53—C54	176.6 (4)
C2—C3—C4—C5	1.7 (7)	C50—N16—C53—C54	−1.9 (7)
C2—C3—C4—C7	179.5 (4)	C49—N16—C53—C58	−1.9 (4)
C3—C4—C5—C6	−1.5 (7)	C50—N16—C53—C58	179.6 (4)
C7—C4—C5—C6	−179.3 (4)	C58—C53—C54—C55	−1.3 (7)
C2—C1—C6—C5	−0.2 (9)	N16—C53—C54—C55	−179.6 (4)
C4—C5—C6—C1	0.8 (8)	C53—C54—C55—C56	−0.9 (7)
C8—N7—C7—C4	−57.4 (4)	C54—C55—C56—C57	1.3 (8)
C19—N7—C7—C4	67.7 (4)	C55—C56—C57—C58	0.7 (7)
Cd1—N7—C7—C4	−177.1 (3)	C56—C57—C58—C53	−2.9 (6)
C3—C4—C7—N7	87.4 (5)	C56—C57—C58—N15	177.6 (4)
C5—C4—C7—N7	−94.9 (5)	C54—C53—C58—C57	3.3 (6)
C19—N7—C8—C9	80.2 (4)	N16—C53—C58—C57	−178.0 (4)
C7—N7—C8—C9	−153.8 (3)	C54—C53—C58—N15	−177.1 (4)
Cd1—N7—C8—C9	−32.3 (3)	N16—C53—C58—N15	1.6 (4)
C18—N9—C9—N8	−0.8 (4)	C49—N15—C58—C57	178.8 (4)
Cd1—N9—C9—N8	−166.9 (2)	Cd1—N15—C58—C57	−1.0 (7)
C18—N9—C9—C8	175.0 (3)	C49—N15—C58—C53	−0.7 (4)
Cd1—N9—C9—C8	8.8 (4)	Cd1—N15—C58—C53	179.5 (3)
C13—N8—C9—N9	1.5 (4)	O1—C59—C60—C61	−170.5 (5)
C10—N8—C9—N9	−177.1 (3)	C64—C59—C60—C61	10.1 (6)
C13—N8—C9—C8	−174.3 (3)	O1—C59—C60—N1	10.2 (7)
C10—N8—C9—C8	7.0 (6)	C64—C59—C60—N1	−169.2 (4)
N7—C8—C9—N9	18.9 (5)	O2—N1—C60—C61	152.5 (5)
N7—C8—C9—N8	−165.7 (3)	O3—N1—C60—C61	−26.6 (7)
C9—N8—C10—C11	−90.1 (5)	O2—N1—C60—C59	−28.1 (8)
C13—N8—C10—C11	91.5 (5)	O3—N1—C60—C59	152.8 (5)
N8—C10—C11—C12	130.7 (5)	C59—C60—C61—C62	−3.2 (7)
C9—N8—C13—C14	177.6 (4)	N1—C60—C61—C62	176.2 (4)
C10—N8—C13—C14	−3.7 (6)	C60—C61—C62—C63	−5.7 (7)
C9—N8—C13—C18	−1.6 (4)	C60—C61—C62—N2	172.8 (4)
C10—N8—C13—C18	177.1 (3)	O5—N2—C62—C61	171.5 (4)
N8—C13—C14—C15	−179.1 (4)	O4—N2—C62—C61	−9.3 (6)
C18—C13—C14—C15	0.0 (6)	O5—N2—C62—C63	−9.9 (6)
C13—C14—C15—C16	−0.3 (6)	O4—N2—C62—C63	169.3 (4)
C14—C15—C16—C17	−0.2 (7)	C61—C62—C63—C64	6.2 (7)
C15—C16—C17—C18	1.1 (6)	N2—C62—C63—C64	−172.4 (4)
C16—C17—C18—C13	−1.4 (6)	C62—C63—C64—N3	179.0 (4)
C16—C17—C18—N9	177.4 (4)	C62—C63—C64—C59	2.1 (7)
N8—C13—C18—C17	−179.8 (3)	O6—N3—C64—C63	3.6 (9)
C14—C13—C18—C17	0.9 (6)	O7—N3—C64—C63	−175.3 (5)
N8—C13—C18—N9	1.2 (4)	O6—N3—C64—C59	−179.4 (7)
C14—C13—C18—N9	−178.1 (3)	O7—N3—C64—C59	1.7 (8)
C9—N9—C18—C17	−179.2 (4)	O1—C59—C64—C63	171.0 (4)
Cd1—N9—C18—C17	−16.8 (6)	C60—C59—C64—C63	−9.6 (6)
C9—N9—C18—C13	−0.3 (4)	O1—C59—C64—N3	−5.8 (7)
Cd1—N9—C18—C13	162.1 (3)	C60—C59—C64—N3	173.6 (4)
C8—N7—C19—C20	−153.8 (3)	O9—N4—C70—C69	−164.1 (4)
C7—N7—C19—C20	80.1 (4)	O10—N4—C70—C69	15.5 (6)
Cd1—N7—C19—C20	−39.1 (3)	O9—N4—C70—C65	20.5 (6)
C29—N11—C20—N10	−0.3 (4)	O10—N4—C70—C65	−159.9 (4)
Cd1—N11—C20—N10	174.3 (2)	O8—C65—C70—C69	−166.8 (4)
C29—N11—C20—C19	175.0 (3)	C66—C65—C70—C69	9.4 (6)
Cd1—N11—C20—C19	−10.4 (5)	O8—C65—C70—N4	8.3 (6)
C24—N10—C20—N11	0.8 (4)	C66—C65—C70—N4	−175.5 (4)
C21—N10—C20—N11	−177.4 (4)	N4—C70—C69—C68	179.5 (4)
C24—N10—C20—C19	−174.4 (4)	C65—C70—C69—C68	−5.3 (6)
C21—N10—C20—C19	7.3 (6)	C70—C69—C68—C67	−1.3 (6)
N7—C19—C20—N11	37.6 (5)	C70—C69—C68—N5	175.6 (4)
N7—C19—C20—N10	−147.7 (3)	O11—N5—C68—C69	−4.9 (6)
C20—N10—C21—C22	103.4 (5)	O12—N5—C68—C69	175.8 (4)
C24—N10—C21—C22	−74.5 (5)	O11—N5—C68—C67	172.0 (4)
N10—C21—C22—C23	−145.9 (7)	O12—N5—C68—C67	−7.2 (6)
C20—N10—C24—C25	179.5 (4)	C69—C68—C67—C66	2.7 (6)
C21—N10—C24—C25	−2.2 (7)	N5—C68—C67—C66	−174.2 (4)
C20—N10—C24—C29	−0.9 (4)	C68—C67—C66—C65	2.5 (7)
C21—N10—C24—C29	177.4 (4)	C68—C67—C66—N6	178.6 (4)
N10—C24—C25—C26	178.7 (4)	O8—C65—C66—C67	168.2 (4)
C29—C24—C25—C26	−0.9 (7)	C70—C65—C66—C67	−8.0 (6)
C24—C25—C26—C27	−0.3 (8)	O8—C65—C66—N6	−7.7 (7)
C25—C26—C27—C28	0.5 (8)	C70—C65—C66—N6	176.1 (4)
C26—C27—C28—C29	0.6 (8)	O14—N6—C66—C67	156.6 (5)
C27—C28—C29—N11	−179.8 (4)	O13—N6—C66—C67	−25.3 (7)
C27—C28—C29—C24	−1.8 (7)	O14—N6—C66—C65	−27.1 (7)
C20—N11—C29—C28	177.9 (5)	O13—N6—C66—C65	150.9 (6)

### Hydrogen-bond geometry (Å, °)

**Table:**

*D*—H···*A*	*D*—H	H···*A*	*D*···*A*	*D*—H···*A*
C3—H3···O5^i^	0.93	2.49	3.265 (6)	140
C8—H8A···O10^ii^	0.97	2.60	3.532 (6)	162
C8—H8B···O4^i^	0.97	2.40	3.363 (6)	171
C21—H21A···O8^ii^	0.97	2.39	3.277 (7)	151
C21—H21B···O8	0.97	2.38	3.110 (7)	132
C37—H37A···O1^iii^	0.97	2.51	3.463 (5)	168
C39—H39B···O1^iii^	0.97	2.40	3.357 (7)	168
C39—H39B···O2^iii^	0.97	2.47	3.101 (8)	123
C50—H50A···O7^iii^	0.97	2.51	3.472 (7)	172
C50—H50B···O1	0.97	2.28	3.109 (7)	142

Symmetry codes: (i) *x*−1, *y*, *z*; (ii) −*x*+1, −*y*, −*z*+1; (iii) −*x*+1, −*y*+1, −*z*.
